# Genetic Mapping in Autohexaploid Sweet Potato with Low-Coverage NGS-Based Genotyping Data

**DOI:** 10.1534/g3.120.401433

**Published:** 2020-06-01

**Authors:** Eiji Yamamoto, Kenta Shirasawa, Takumi Kimura, Yuki Monden, Masaru Tanaka, Sachiko Isobe

**Affiliations:** * Kazusa DNA Research Institute, Japan,; ^†^ Graduate School of Environmental and Life Science, Okayama University, Japan, and; ^‡^ Kyushu Okinawa Agricultural Research Center, National Agriculture and Food Research Organization, Japan

**Keywords:** Allele dosage, Autopolyploid, Genetic mapping, NGS-based genotyping, Sweet potato

## Abstract

Next-generation sequencing (NGS)-based genotyping methods can generate numerous genetic markers in a single experiment and have contributed to plant genetic mapping. However, for high precision genetic analysis, the complicated genetic segregation mode in polyploid organisms requires high-coverage NGS data and elaborate analytical algorithms. In the present study, we propose a simple strategy for the genetic mapping of polyploids using low-coverage NGS data. The validity of the strategy was investigated using simulated data. Previous studies indicated that accurate allele dosage estimation from low-coverage NGS data (read depth < 40) is difficult. Therefore, we used allele dosage probabilities calculated from read counts in association analyses to detect loci associated with phenotypic variations. The allele dosage probabilities showed significant detection power, although higher allele dosage estimation accuracy resulted in higher detection power. On the contrary, differences in the segregation patterns between the marker and causal genes resulted in a drastic decrease in detection power even if the marker and casual genes were in complete linkage and the allele dosage estimation was accurate. These results indicated that the use of a larger number of markers is advantageous, even if the accuracy of allele dosage estimation is low. Finally, we applied the strategy for the genetic mapping of autohexaploid sweet potato (*Ipomoea batatas*) populations to detect loci associated with agronomic traits. Our strategy could constitute a cost-effective approach for preliminary experiments done performed to large-scale studies.

Recent advances in next-generation sequencing (NGS) technology have revolutionized genomics-assisted breeding. NGS-based genotyping by sequencing (GBS; Elshire *et al.* 2011) and restriction site-associated DNA sequencing (RAD-seq; Baird *et al.* 2008) have enabled the development of numerous genetic markers in a single experiment (Kumar *et al.* 2012). They have been used to construct high-density genetic linkage maps (Poland and Rife 2012) and genetic maps of agronomically important traits. These technologies are highly effective with diploid species; however, they present numerous application challenges with autopolyploid species (Bourke *et al.* 2018).

Polyploidy is the presence of multiple sets of chromosomes in a single organism and is a common occurrence in the plant kingdom. Polyploid plant species are often valuable as crops, as their genome multiplication results in comparatively higher yields (Comai 2005). In addition, polyploidy often leads to heterosis, gene redundancy, loss of self-incompatibility, and gains in asexual reproduction (Comai 2005). In allopolyploid species, such as cotton and wheat, preferential pairing dictates meiotic chromosome behavior similar to diploids. As this mechanism resembles that seen in diploids, currently available genetic approaches can be readily applied to allopolyploids. By contrast, autopolyploids have multiple heterozygous genotypes. Consequently, the existing approaches designed for diploids are not applicable to autopolyploids (Bourke *et al.* 2018). A possible solution for this problem is the use of Mendelian markers such as Simplex × Nullplex (SN) and Simplex × Simplex (SS). The mode of inheritance of Mendelian markers resembles that for the genetic markers in diploid species. Thus, they apply to the theories and/or tools developed for diploids (Shirasawa *et al.* 2017; Tennessen *et al.* 2014; Vukosavljev *et al.* 2016). To detect genetic loci with a simple inheritance and/or a high proportion of the variance explained, the use of Mendelian markers alone may suffice. However, genetic mapping based on allele dosage information may be required for more complex phenotypes (Rosyara *et al.* 2016).

To use multiple-dose markers, the allele dosage must be determined. Several techniques can be used to estimate allele dosage in polyploids (Clark *et al.* 2019; Gerard *et al.* 2018; Gerard and Ferrão 2020; Serang *et al.* 2012; Wadl *et al.* 2018). These techniques have enabled the development of genetic mapping methods for polyploids (da Silva Pereira *et al.* 2019; Rosyara *et al.* 2016). Even with the available tools, accurate allele dosage estimation demands an adequate amount of high-quality data. To meet this requirement, the first allele dosage estimation method was developed for SNP-chip data (Serang *et al.* 2012). For NGS-based genotyping, abundant sequence data are needed for species at higher ploidy levels and with larger genome sizes. Gerard *et al.* (2018) recommended read depths > 25 and > 90 to obtain accurate allele dosages for autotetraploids and autohexaploids, respectively. Wadl *et al.* (2018) developed a GBS pipeline for polyploid study. They reported that > 100 reads were necessary to achieve 95% accuracy for allele dosage estimation in autohexaploid species.

The main objective of this study was to perform genetic mapping in polyploids in a cost-effective manner (*i.e.*, with low-coverage NGS-based genotyping data). We propose a simple genetic mapping strategy for autopolyploids using low-coverage NGS data, and evaluate its validity using simulated and real data from two genetic mapping populations in sweet potato (*Ipomoea batatas* (L.) Lam). Sweet potato is a hexaploid species with 90 chromosomes (2n = 6x = 90). In our proposed method, the allele dosage probability for each single-nucleotide polymorphism (SNP) marker site is calculated on the basis of read depth information from low-coverage double digest (dd) RAD-seq genotyping data. We did not attempt to determine allele dosage where the read depths were too small. Alternatively, allele dosage probabilities can be used in subsequent genetic mappings. This idea is similar to a previous study that used continuous genotype values from the signal intensities of the SNP-chips for genetic mapping (Grandke *et al.* 2016). In the present study, we applied this idea to low-coverage NGS-based genotyping data. In this manner, the maximum use of the available genetic marker information can be made.

## Materials and Methods

### Plant materials and phenotypes

**Figure 1:**
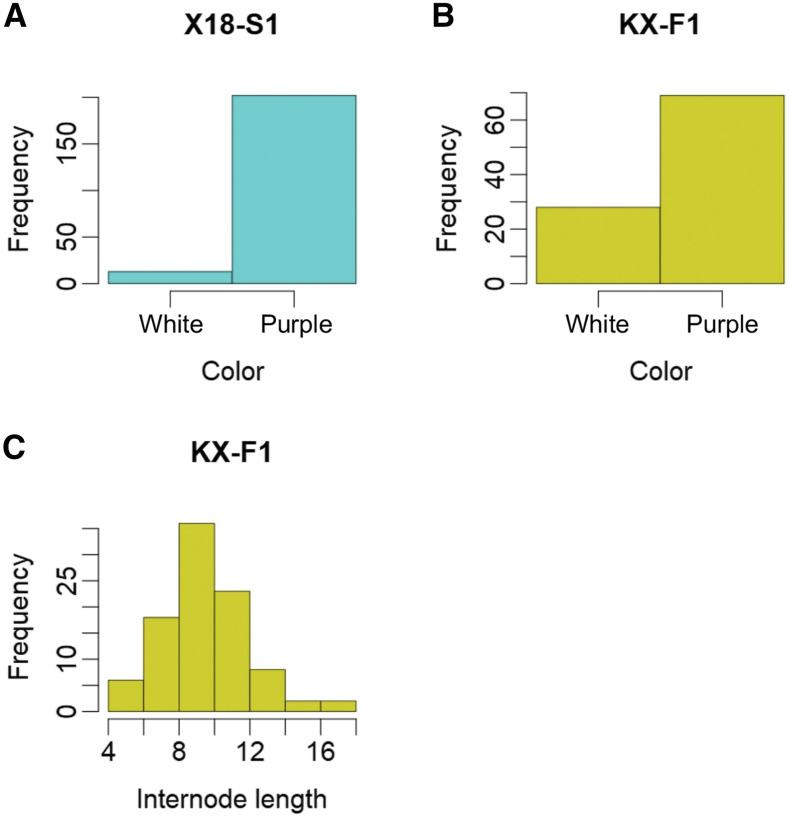
Distribution of phenotypic values. (A) Color of X18-S1. (B) Color of KX-F1. (C) Internode length of KX-F1.

Two populations of autohexaploid sweet potato (2n = 6x = 90) were used. One was the F_1_ derived from reciprocal crosses between the major Chinese variety Xushu 18 and the wild sweet potato (*Ipomoea trifida*) (K123–11); hereafter, this population is referred to as KX-F1. The other population originated from self-pollinated (S_1_) Xushu 18 (n = 248) used in a previous study (Shirasawa *et al.* 2017); hereafter, this population is called X18-S1. These materials were developed by the Kyushu Okinawa Agricultural Research Center of the National Agriculture and Food Research Organization (KARC/NARO). KX-F1 was phenotyped for color and internode length, and X18-S1 was phenotyped only for color (Table 1, Figure 1). KX-F1 was planted in a field at Okayama University from June through to November 2016. X18-S1 was planted in a field at the Miyakonojo Research Station of KARC/NARO, from June through to November 2016.

**Table 1:** Populations and traits analyzed in the present study

Population	Trait	Details
KX-F1 (*n* = 99)	Color	Skin color: 0 and 1 for white and purple, respectively.
	Internode length	Average internode length (cm).
X18-S1 (*n* = 248)	Color	Skin color: 0 and 1 for white and purple, respectively.

### ddRAD-seq and variant calling

Genomic DNA was extracted from the KX-F1 leaves with a DNeasy Plant Mini Kit (Qiagen, Hilden, Germany). The ddRAD-seq analyses were performed as described in Shirasawa *et al.* (2016), and ddRAD-Seq libraries were constructed using the restriction enzymes *Pst*I and *Msp*I. DNA fragments of 300–900 bp in length were fractionated using BluePippin (Sage Science, Beverly, MA, USA). The nucleotide sequences of the libraries were determined on the HiSeq 2000 and HiSeq 4000 platforms (Illumina, San Diego, CA, USA) in paired-end mode (93 base or 101 base). The ddRAD-seq reads for the X18-S1 populations were obtained from the DNA Data Bank of Japan (DDBJ) sequence archive, under the accession numbers DRA004836, DRA004837, and DRA004838. Data were processed as described in Shirasawa *et al.* (2017). Low-quality sequences were removed and adapters were trimmed with PRINSEQ v. 0.20.4 (Schmieder and Edwards 2011) and fastx_clipper in the FASTX-Toolkit v. 0.0.13 (http://hannonlab.cshl.edu/fastx_toolkit). The filtered reads were mapped onto the *I*. *trifida* “Mx23Hm” (ITR_r1.0) genome sequence (Hirakawa *et al.* 2015) using Bowtie 2 v. 2.2.3 (Langmead and Salzberg 2012). The parameters were set as the maximum fragment size length (I) = 1000 and the ‘–sensitive’ preset of the ‘–end-to-end’ mode. The sequence alignment/map (SAM) files were converted into binary sequence alignment/map (BAM) files and subjected to SNP calling, using the mpileup option in SAMtools v. 0.1.19 (Li *et al.* 2009) and the mpileup2snp option of VarScan 2 v. 2.3 (Koboldt *et al.* 2012). The output was a VCF file with SNP data. The information in the VCF files was loaded into the R platform via ‘read.vcfR’ in *vcfR* (Knaus and Grünwald 2017). Markers with missing values > 0.5 were filtered out using an in-house R script (R Core Team 2018).

### Allele dosage estimation

**Figure 2:**
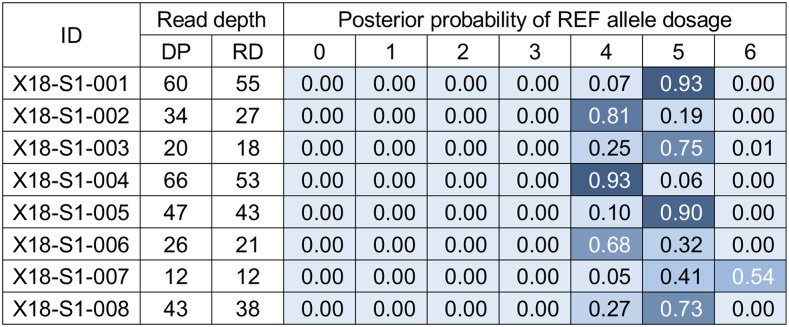
Example of allele dosage estimation. Part of the result at SNP Itr_sc000001.1_24872 in X18-S1. ID = the name of individuals. DP and RD in the read depth column mean the total and reference genome type read depths, respectively. Values in the colored cells indicate the relative allele dosage probabilities. The estimation was performed using the “S1” method in updog.

In the present study, the allele dosage was the dosage of the reference genome type allele for each SNP locus. Results of allele dosage estimation were represented by a matrix consisting of probability values (Figure 2). For allele dosage estimation, information on the depths of the total (DP) and the reference type (RD) reads were extracted from the VCF files using ‘extract.gt’ in *vcfR* of R (Knaus and Grünwald 2017). Individual genotypes with DP < 10 and DP > 300 were filtered out from further analyses. The SNP markers included potential monomorphic markers. These were identified using a major genotype frequency, namely, the ratio of individuals with a specific allele dosage at a given SNP marker. The major genotype frequency (MGF) was estimated by aggregating the column elements of the allele dosage matrix (Figure 2). MGF > 0.95 were filtered out. Gerard *et al.* (2018) reported noise factors that disturb allele dosage estimation from the NGS data. Allelic bias represents the differences in the detectability between alleles due to experimental constraints, such as difficulties in the detection of certain sequences. Overdispersion is an additional variability from the expected data appearance patterns in the observations. This phenomenon is explained by the differences in the experimental noises between samples. In the present study for allele dosage estimation we used the ‘multidog’ function in the R package *updog*, which considers those noise factors (Gerard *et al.* 2018; Gerard and Ferrão 2020). The ‘S1’ and ‘F1’ options were used for the S1 and F1 populations, respectively. The ‘Norm’ option, which is the recommendation of the function, was also used for the simulation experiments.

This method is powerful and accurate, its only drawback being the computational time. As an alternative, we used the following naïve method. For N-ploid species, the possible allele dosage states are *d* ∈ {0/N, 1/N, …, N/N}. For real data, errors in the experimental procedure introduce bias relative to the theoretical probabilities. Therefore, the expected value of the allele dosage *d* in the real data were determined as *r* ∈ {0/N+e, 1/N, …, N/N-e}, where e is the unknown error probability. We used an ad hoc error probability of 0.001, because this value resulted in a shorter computational time. For a given DP and RD, the probability (Pr) of dosage *d_i_* was calculated using the binomial distribution function:

Pr(Dosage=di)=CDPRD×riRD×(1−ri)DP−RD (1)

Thus, N + 1 probability values were calculated for each individual at each SNP site. The relative probability (RPr) for the allele dosage *d_i_* was calculated as follows:

$$\text{RPr}\left(\text{Dosage}=d_{i}\right)=\text{Pr}\left(\text{Dosage}=d_{i}\right)/\underset{i}{\sum }\text{Pr}(\text{Dosage}=d_{i})$$ (2)

In this way, a matrix *M* was obtained for each SNP marker, with individuals as row elements. The column elements were the relative probabilities of the reference type allele dosages calculated by equation (2). Calculation with the binomial distribution function was performed in ‘dbinom’ in R (R Core Team 2018). We revisit the validity of this naïve method in the results and discussion.

### Association analyses

The association between marker genotype and phenotype was tested with a generalized linear model (GLM) using

$$\hat{y}=\beta_{0}+\beta_{1}x$$ (3)

for continuous traits, and

$$\hat{\pi }=1/\left\{1+exp\left(-\left(\beta_{0}+\beta_{1}x\right)\right)\right\}$$ (4)

The term $\hat{y}$ is a vector of estimated phenotypic values from GLM. $\hat{\pi }$ is equal to Pr{Binary trait value = 1} (where 1 denotes purple, and 0 denotes white in the present study). $\beta_{0}$ is the intercept, $\beta_{1}$ is a vector for the effects of each allele dosage state, and x is the estimated allele dosage information on each SNP marker. To test the statistical significance of each SNP marker, we performed the likelihood ratio test of whether $\beta_{1}$ = 0 or not. GLM fitting was performed using ‘glm’ in R (R Core Team 2018). The augment family functions ‘binomial’ and ‘gaussian’ were used for binary and continuous traits, respectively. The likelihood ratio test was performed using ‘pchisq’ in R (R Core Team 2018) with deviance and degrees of freedom from each GLM as the arguments. For the estimated allele dosage information (x in equation 3 and 4), we used two methods. The first method was the continuous allele dosage that was the product of a matrix of RPr allele dosage and a vector of possible allele dosage (*v* = {0, 1, 2, 3, 4, 5, 6}) (Continuous). This method was analogous to the one used in Grandke *et al.* (2016). In the second method, the matrix of allele dosage information was directly used as *x* in the equations (3) and (4) (Dogmat). This approach was analogous to the ‘general’ option, from a feature of the R package *GWASpoly* (Rosyara *et al.* 2016), but the method from our study did not use the kinship matrix for the covariate of the test.

The genome-wide significant threshold was determined using a permutation test with 1000 replications. A low number of replications often results in the underestimation of the significance thresholds, and it has been suggested that estimating thresholds by using a generalized extreme-value model is more efficient than taking empirical quantiles. Therefore, we fit a generalized extreme value by means of the maximum-likelihood method to the values obtained from the simulations using R package *evd* (Stephenson 2002) (Table 2).

**Table 2:** Estimated genome-wide significance thresholds. The values are −log_10_(*p*) obtained from 1,000 permutation tests

Population	Trait	Association analysis method	Alpha-level		
			10%	5%	1%
X18-S1	Simulated phenotype	Continuous	5.54	5.86	6.62
X18-S1	Simulated phenotype	Dogmat	5.42	5.76	6.46
X18-S1	Color	Continuous	6.36	6.71	7.52
X18-S1	Color	Dogmat	7.00	7.33	8.30
KX-F1	Color	Continuous	6.03	6.37	7.14
KX-F1	Color	Dogmat	7.74	8.35	9.73
KX-F1	Internode length	Continuous	5.76	6.21	6.65
KX-F1	Internode length	Dogmat	6.71	7.05	7.82

To build Manhattan plots of the association analyses, the SNP markers were allocated to 15 homologous linkage groups identified in a previous study (Shirasawa *et al.* 2017). Note that the order of the SNP markers in a homologous linkage group did not correspond to a genetic or physical map position, because the genetic map of Shirasawa *et al.* (2017) did not include SNP marker information, except for the SS markers, and the physical map of the reference genome (Hirakawa *et al.* 2015) used in the present study was fragmented. The Manhattan plot was created with ‘manhattan’ in R package *qqman* (Turner 2014).

### Simulation conditions

To investigate the validity and the power of the proposed strategy, we performed simulation experiments. In these simulations we assumed the genetic analyses of the S_1_ population in an autohexaploid species. The genetic segregation pattern of a locus in the simulated population was Simplex × Simplex (SS), Duplex × Duplex (DD) or Triplex × Triplex (TT). The simulated genotype and NGS data were generated using the ‘rgeno’ and ‘rflexdog’ functions from the R package *updog*, respectively (Gerard *et al.* 2018). We prepared two conditions; one with no allelic bias and overdispersion in the NGS data (option od = 0 and bias = 1, in ‘rflexdog’), and another with allelic bias and overdispersion in the NGS data (option od = 0.05 and bias = 0.5, in ‘rflexdog’).

To evaluate the accuracy of allele dosage estimation, we used a proportion of individuals with correctly determined allele dosage. Since these estimations consisted of probabilistic values (Figure 2), we determined allele dosage as the dosage that showed the highest probability. For example, in the case of X18-S1-003 the allele dosage was ‘5′ (Figure 2).

For the simplicity of the experimental design and for the understandability of the results we assumed that the segregation of a single locus was associated with the simulated phenotype. In general it is difficult to generalize the power needed to detect an association between a marker and a phenotypic variation, as this depends on the population size and the set of markers used in the analysis. In the simulation experiment we used two settings to bring the condition closer to reality, and to make the results easier to understand. First, in the present study the markers and genes that affect phenotypic variation were created by simulation and not selected from real genetic markers. This was necessary to determine the genetic effect of the simulated phenotype, because the true allele dosage was not available in the low coverage NGS data. Second, all real genetic markers in the X18-S1 population were used as background genetic markers that were not associated with the simulated phenotype. This setting was used to determine the significant threshold for the association study.

**Figure 3:**
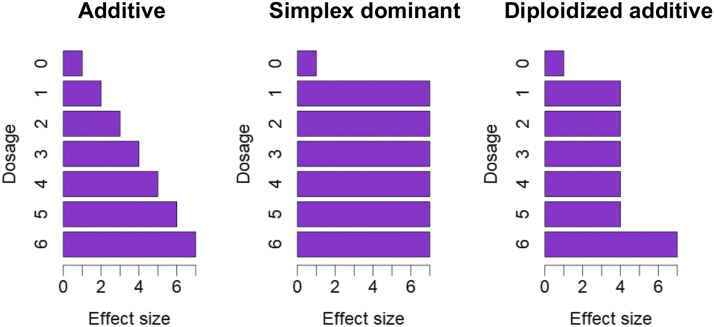
Schematic representation of genetic effect models used for simulations in the present study.

The complicated genetic segregation mode in polyploid species results in various possible genetic effect models (Rosyara *et al.* 2016). In the present study, we simulated three genetic effect models that were analogous to models from a previous study (Rosyara *et al.* 2016) (Figure 3). In the additive model, the genetic effect was proportional to the allele dosage. In the simplex dominant model, all heterozygotes were equivalent to one of the homozygotes. In the diploidized additive model, all heterozygotes were equivalent, and exactly halfway between the two homozygotes.

The proportion of the phenotypic variance explained by a gene (PVE) strongly affected the power to detect the gene. In the present study, we simulated genes with PVE values of 0.1, 0.2, and 0.3. We used the ‘optimize’ function of R to adjust the phenotypes to the PVE.

To investigate the relationship between the power to detect and the genetic distance between the marker and the target gene, the number of crossovers between the marker and the gene was determined using a random variable drawn from a Poisson distribution. The lambda parameter of the Poisson distribution (*i.e.*, the expected value of the random variable) was set as the length of the genetic map distance (in cM) between the marker and the gene. The number of crossovers for each simulation experiment was drawn from the ‘rpois’ function in R.

### Data availability

The phenotype and genotype data, the R scripts, and the R package developed for the present study are available at https://github.com/yame-repos/ngsAssocPoly and archived at DOI: 10.5281/zenodo.3861960. The sequence data from the ddRAD-seq libraries are available in the DDBJ sequence read archive under accession numbers DRA004836–DRA004838 and DRA008654–DRA008655 for X18-S1 and KX-F1, respectively.

## Results

### Allele dosage estimation in low-coverage NGS data

**Figure 4:**
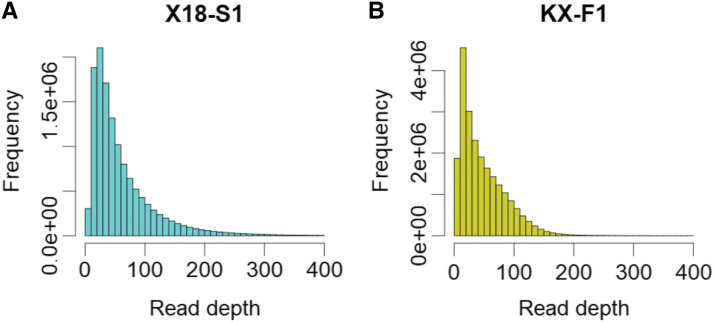
Distribution of read depths for each individual on an SNP marker site. (A) Read depths of X18-S1. (B) Read depths of KX-F1.

**Figure 5:**
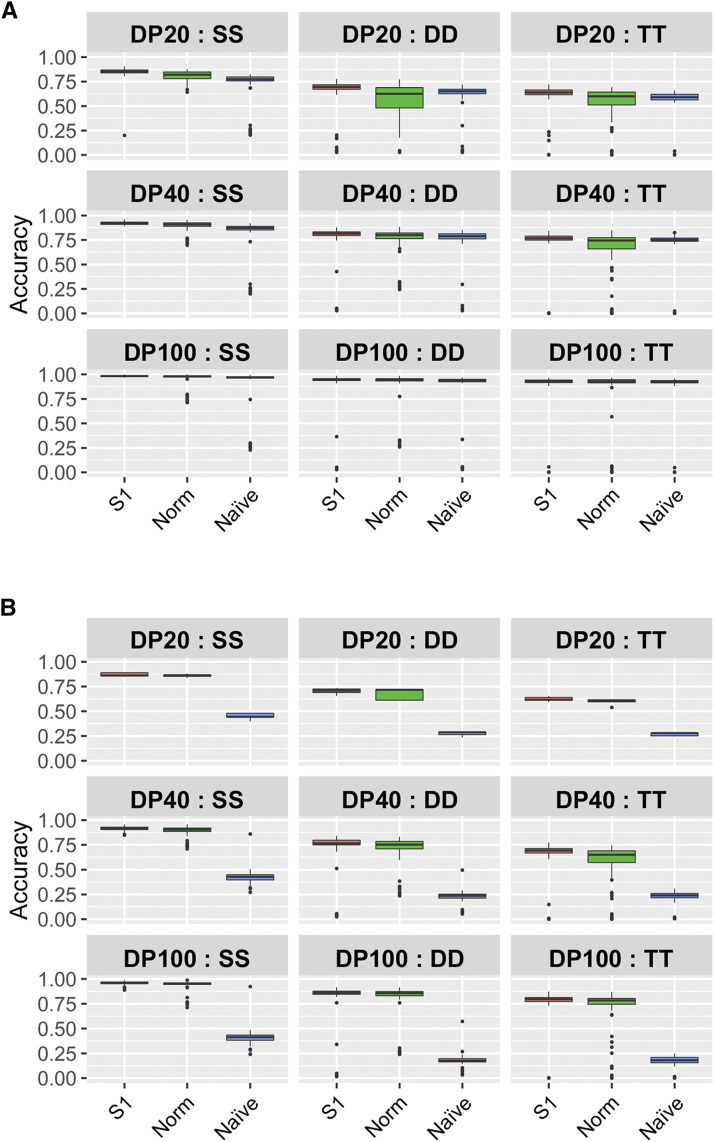
Accuracy of allele dosage estimation using low-coverage NGS data. Accuracy is indicated by the proportion of individuals with correctly estimated allele dosage. The values are based on 100 random simulations. DP indicates read depths. SS, DD and TT indicate Simplex × Simplex, Duplex × Duplex and Triplex × Triplex markers, respectively. Results obtained without (A) and with (B) allelic bias and overdispersion in the NGS data.

The ddRAD-seq data from autohexaploid sweet potato populations was obtained with a standard protocol used for diploid populations (Shirasawa *et al.* 2017). Figure 4 shows the distribution of the read depths. In both X18-S1 and KX-F1, the mode was about 20, and the medians were 37 and 44, respectively. In previous studies, for the accurate estimation of allele dosage read depth of over 100 was recommended (Gerard *et al.* 2018; Wadl *et al.* 2018). Therefore, to investigate the accuracy of allele dosage estimation in low-coverage data (DP = 20 and 40) we performed simulation experiments, and compared the results with the recommended read depth (DP = 100). As expected, higher read depths resulted in higher accuracy (Figure 5). For the allele dosage estimation methods, the inclusion of prior information on the population type (“S1” in Figure 5) and/or consideration of noises in the NGS data (“S1” and “Norm” in Figure 5) resulted in higher accuracy than the Naïve method (Naïve in Figure 5) when the NGS data included noises (Figure 5B). However, the accuracy of allele dosage estimation in markers with complicated segregation mode (*i.e.*, DD and TT in Figure 5) was less than 0.75 in the 20 read depth data, even if the estimation method considered noises in the NGS data (Figure 5). These results confirmed the conclusions of previous studies, namely that for an accurate allele dosage estimation high-coverage NGS data are necessary, and thus it is difficult to use low-coverage NGS data for some standard genetics approaches, such as the construction of linkage map and QTL interval mapping (da Silva Pereira *et al.* 2019; Mollinari and Garcia 2019, 2020).

### Potential assessment of genetic mapping using low-coverage NGS data

**Figure 6:**
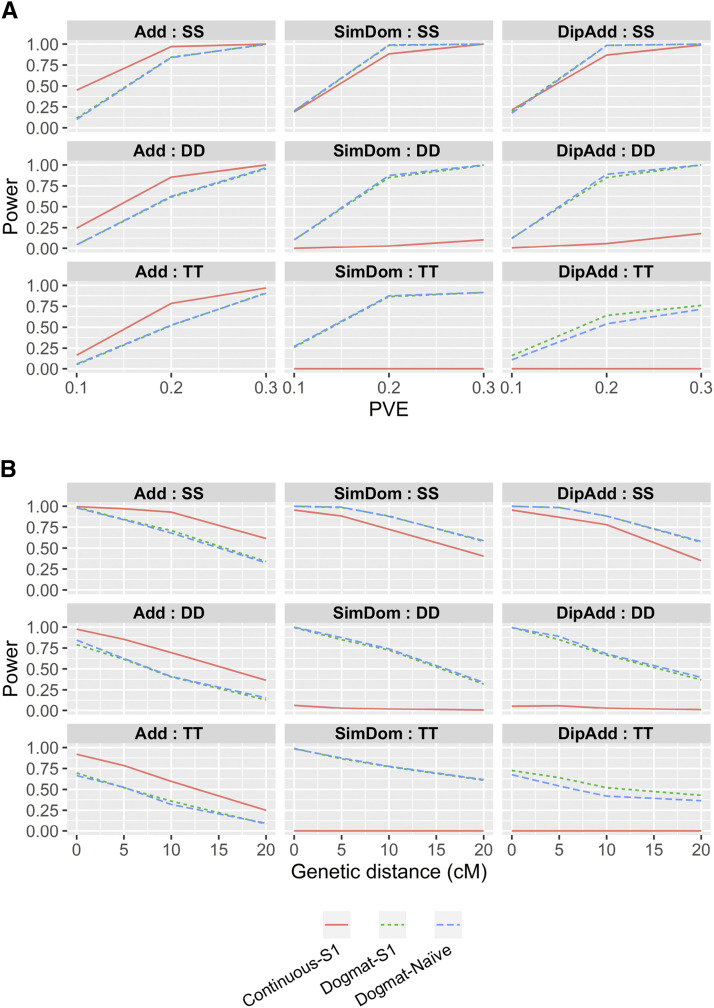
Investigation of the power to detect associations between genetic markers and phenotypic variation. Power indicates the proportion of experiments that achieved higher −log_10_(*p*) value than the significant threshold value determined by permutation test. Results were obtained from 100 random simulations. Read depths in the simulated markers were 20 and 40. SS, DD, and TT indicate Simplex × Simplex, Duplex × Duplex, and Triplex × Triplex markers, respectively. Add, SimDom, and DipAdd indicate additive, simplex dominant, and diploidized additive, respectively. “Continuous” and “Dogmat” indicate continuous allele dosage values and matrix of allele dosage, respectively. S1 and Naïve indicate methods to estimate allele dosage. (A) Relationships between the power and proportion of variance are explained by the target gene (PVE). Genetic distance from marker to target gene was 5 cM. (B) Relationships between the power and genetic distance from marker to target gene. PVE of the simulated gene was 0.2.

**Figure 7:**
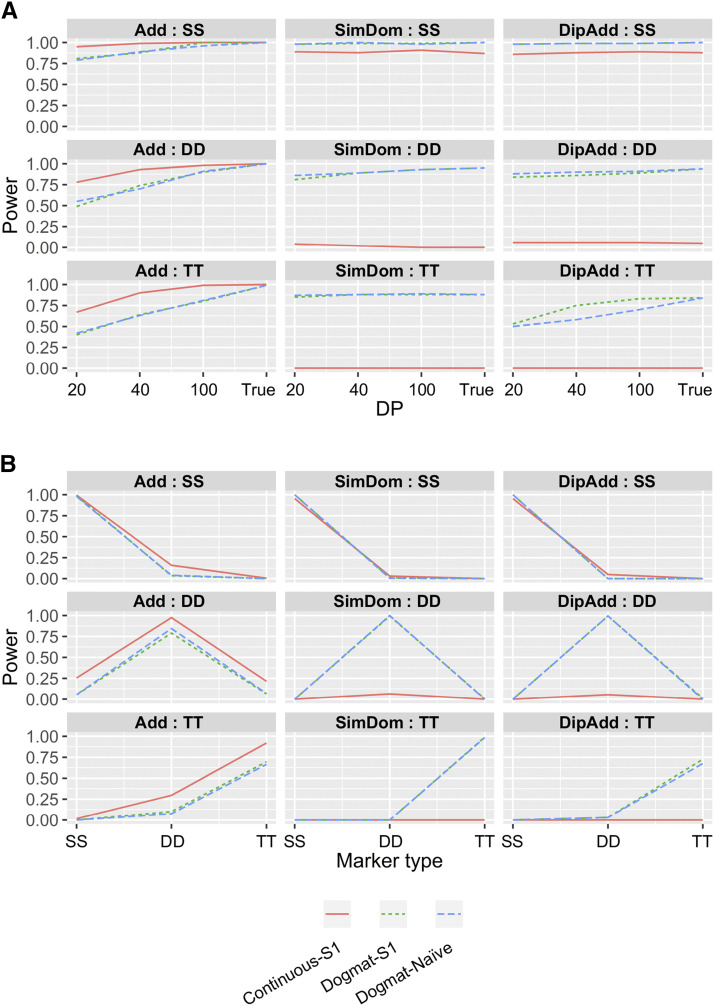
Investigation of the power to detect associations between genetic markers and phenotypic variation under the conditions specific to polyploids. Power indicates the proportion of experiments that achieved higher −log_10_(*p*) value than the significant threshold value determined by permutation test. The values are based on 100 random simulations. PVE of the simulated gene was 0.2. SS, DD, and TT indicate Simplex × Simplex, Duplex × Duplex, and Triplex × Triplex markers, respectively. Add, SimDom, and DipAdd indicate additive, simplex dominant, and diploidized additive, respectively. “Continuous” and “Dogmat” indicate continuous allele dosage values and matrix of allele dosage, respectively. S1 and Naïve indicate methods to estimate allele dosage. (A) Relationship between the power and read depths (DP). True indicates the cases where true allele dosage information was available. Genetic distance from marker to target gene was 5 cM. (B) Relationships between the power and the genetic segregation patterns of the markers and the target gene. SS, DD or TT on the title of each panel indicate the segregation patterns of the target gene. Marker type on the *x*-axes indicates the segregation pattern of markers. Read depths in the simulated markers were 20 and 40. Genetic distance from marker to target gene was 0 cM.

Although the determination of allele dosage from low-coverage NGS data were difficult (Figure 5), a previous study reported that unspecified continuous allele dosage values were applicable to genetic mapping (Grandke *et al.* 2016). Using simulation experiments, we investigated whether the probability information could be useful for genetic mapping (Figure 2). Power increased as the PVE of the gene increased (Figure 6A), and it decreased when the distance between the marker and target genes became larger (Figure 6B). Next, we investigated the relationship between the genetic effect models (Figure 3) and read depths (Figure 7A). The estimated allele dosage from the low-coverage NGS data (DP = 20 and 40) showed power to detect association, although the use of the true allele dosage had a higher power (Figure 7A). For an additive genetic effect, the continuous allele dosage (Continuous in Figure 7) showed higher power, while the matrix of allele dosage (Dogmat in Figure 7) showed a higher power for the simplex dominant and the diploidized additive. This feature was prominent when the marker had a complicated segregation pattern (DD and TT in Figure 7A). Interestingly, the power to detect an association between the dosage matrix from updog and the naïve method were not different (S1-Dogmat and Naïve-Dogmat in Figure 7A), despite the significantly higher accuracy of allele dosage estimation in updog (Figure 5).

Differences in the segregation patterns between the marker and the target gene can prevent the detection of a gene even if the marker is on the same genetic region. We investigated the situation where the marker and the target gene were in complete linkage, but their segregation patterns were different (*e.g.*, the gene was SS, but the marker was DD). This difference in the segregation pattern between the marker and the target gene resulted in a drastic decrease in detection power (Figure 7B), indicating that the use of a larger number of markers is more advantageous to detect associations, even if the accuracy of allele dosage estimation is low.

### Genetic mapping of sweet potato agronomic traits

**Figure 8:**
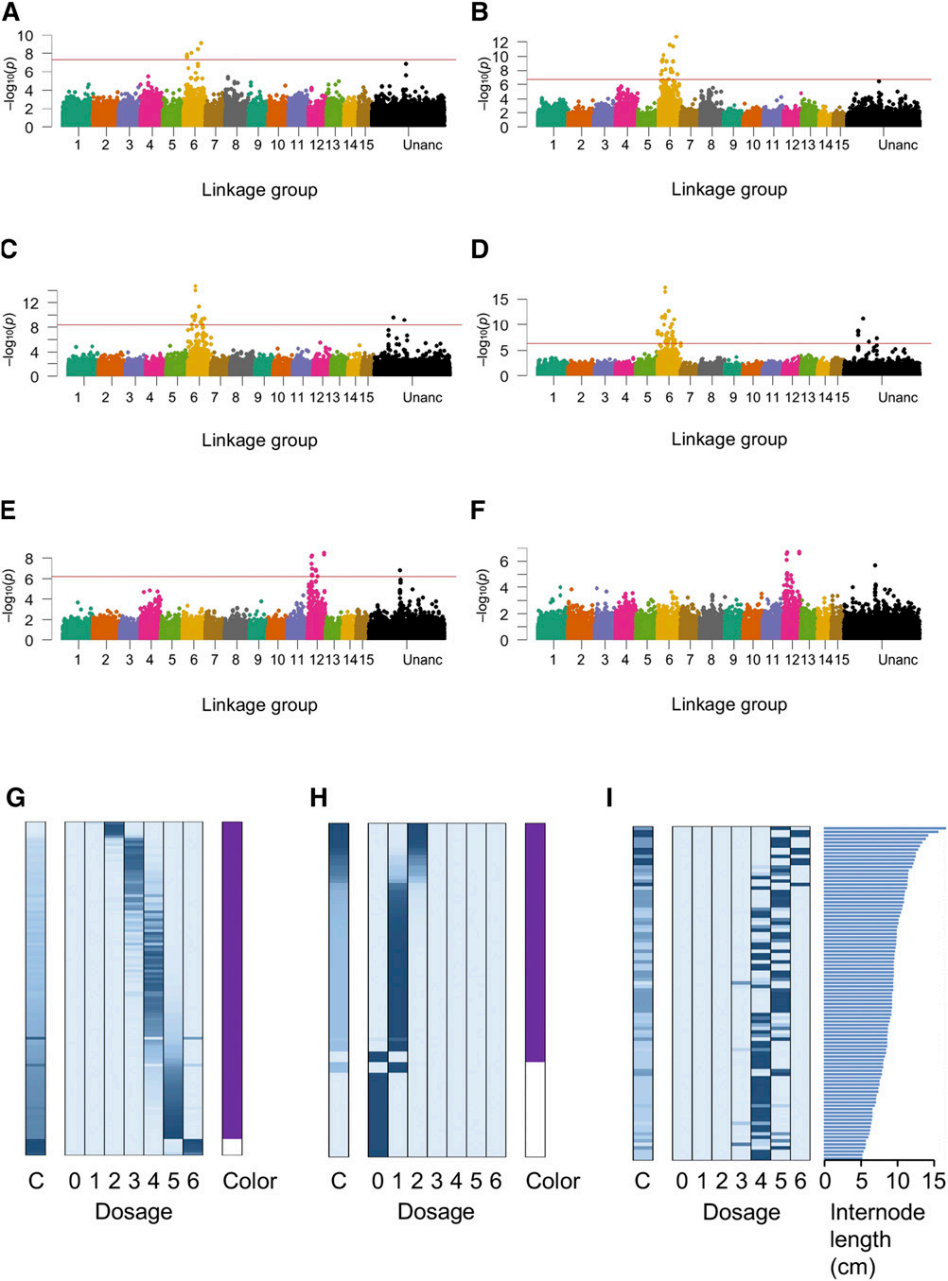
Genetic mapping using real genotype and phenotype data from autohexaploid populations. (A-F) Manhattan plots of the association analyses for agronomic traits. The red lines in the Manhattan plots indicate a 5% genome-wide significance threshold based on 1000 replicates of the permutation test. (A) Color of the X18-S1 using the Continuous method. (B) Color of the X18-S1 using the Dogmat method. (C) Color of the KX-F1 using the Continuous method. (D) Color of the KX-F1, using the Dogmat method. (E) Internode length of KX-F1, using the Continuous method. (F) Internode length of KX-F1 using the Dogmat method. (G-I) Comparisons between the phenotypic values and the allele dosage probabilities for the SNP markers. Boxes in each panel indicate allele dosage probabilities. Rows indicate individuals, and blue cells in the columns indicate individual allele dosage probabilities. The gradient from white to blue indicates values ranging from 0 to 1. Plots on the right side of each panel indicate the phenotype. (G) SNP Itr_sc000723.1_30361 in X18-S1. (H) SNP Itr_sc000236.1_59664 in KX-F1. (I) SNP Itr_sc002801.1_4640 in KX-F1.

We performed association analyses for the real phenotypes in X18-S1 and KX-F1 using the genotype information called by the naïve method (Table 1, Figure 1). Color is a qualitative, binary phenotype, while internode length was a quantitative phenotype. For color, strong significant peaks (Itr_sc000236.1_59664 and Itr_sc000723.1_30361 for KX-F1 and X18-S1, respectively) were detected on homologous group 6 (Figure 8A-D). Comparisons of the phenotypic values and the estimated allele dosages at the significant SNPs indicated that the phenotype inheritance mode was simplex dominant (Figure 8G and H).

For the internode length of KX-F1, a significant peak was detected on homologous group 12 (Figure 8E and F). A comparison between the estimated allele dosage at the highest signal (Itr_sc002801.1_4640) and the internode length phenotype suggested a proportional relationship between the phenotypic value and the allele dosage (Figure 8I), indicating that the genetic effect model was additive (Figure 3). In the simulation experiment, the association analysis with the continuous genotype showed a higher power than the dosage matrix genotype for the gene with the additive genetic effect model (Figure 7A). In fact, the highest signal on internode length showed a higher −log_10_(*P*) value in the continuous genotype than in the dosage matrix genotype (Figure 8E, F).

## Discussion

We genotyped populations using ddRAD-seq. In general, polyploid species have large genomes and multiple allele dosages. Therefore, numerous markers must be developed to capture genetic variation in the entire genomic region. NGS-based genotyping, such as RAD-seq and GBS, are powerful because they generate hundreds of thousands of markers per experiment. High read counts per marker are necessary for the accurate allele dosage estimation (Gerard *et al.* 2018; Wadl *et al.* 2018). The NGS data used in the present study were obtained through a standard protocol that is used for diploid populations (Shirasawa *et al.* 2017). Therefore, the filtration of markers with 100 read depths purged most markers (Figure 4). Hence, we focused on the allele dosage probability, rather than determination, and we used a comparatively larger number of markers as genotype data in our association analyses.

In order to evaluate the accuracy of allele dosage estimation in low-coverage NGS data, we performed simulation experiments, however, the determination of allele dosage with low-coverage data, especially in markers with complicated segregation patterns (DD and TT in Figure 5), was difficult. It has been reported that polyRAD could achieve a higher allele dosage estimation accuracy in low-coverage NGS data by using information on parental genotype and linkage disequilibrium (Clark *et al.* 2019). In the present study, we did not use polyRAD because parental genotype information was not available, and the reference genome was not enough to determine the physical order of markers identified in the present study. Since the genetic mapping of polyploid species is still a developing area in genetics, the use of the latest methods and the surrounding information (such as reference genomes), will improve the accuracy of allele dosage estimation.

Interval mapping that uses estimated genotype information between markers is a powerful approach to detect genetic loci associated with phenotypic variation (da Silva Pereira *et al.* 2019). In the present study, we did not perform an interval mapping approach because the markers identified here were not enough to construct a genetic linkage map. Besides, the objective of our study was to determine if the genetic mapping of a polyploid species could be possible when accurate allele dosage information is not available. We performed a simple marker-phenotype association analysis to detect the genetic loci associated with phenotypic variations. In the simulation experiments, the estimated allele dosage showed enough power to detect an association, although accurate allele dosage information showed higher power (Figure 7A). Interestingly, there was little difference in the power to detect an association between S1 and Naïve (Figure 7), despite the accuracy of allele dosage estimation being apparently higher in S1 than in Naïve (Figure 5). This is probably because of the effect of allelic bias on the estimation of allele dosage, as it has been reported that the allelic bias representing the difference in the observed allele frequency and the true allele frequency had problem in the determination of allele dosage (Gerard *et al.* 2018). On the other hand, the impact of allelic bias on the association analysis will be smaller because in most cases allelic bias results in a proportional shift of the allele dosage values. For this reason, there will be little change in the information content as an explanatory variable of the association analysis. Since the allele dosage estimation methods accounting for noises in the NGS data require a longer computational time than the naïve method, the latter can be used as a good alternative approach when the number of markers are extremely large. Perhaps the most important result of this study is that the difference in the segregation patterns between the marker and the target gene resulted in a drastic decrease in detection power (Figure 7B). This indicates that the use of a larger number of markers is more advantageous to detect associations, even if the accuracy of the allele dosage estimation is low. This conclusion is different from those that used elaborate methods requiring accurate allele dosage information (da Silva Pereira *et al.* 2019; Mollinari *et al.* 2020).

Finally, we confirmed the applicability of the proposed strategy on real genotype and phenotype data from autohexaploid sweet potato (Figure 8). Unlike other tools and methods, the only prerequisite condition in the strategy was that the genotype data must be obtained by NGS-based methods. For this reason, the approach used in the present study was easy to use. Nevertheless, a drawback of this characteristic is that it ignores certain information, such as the precise chromosomal locations of the genes, because it is difficult to construct genetic linkage map with low-coverage NGS data. Elaborate methods that specifically determine allele dosage, are necessary to precisely map the genetic loci (Bourke *et al.* 2018; da Silva Pereira *et al.* 2019; Mollinari and Garcia 2019; Rosyara *et al.* 2016). To use them, however, an abundance of high-quality genotype data are necessary. Therefore, we recommend the following genetic mapping strategy for autopolyploid crop species: (1) Perform NGS-based genotyping using a reasonable data volume. (2) If positive results are obtained, increase the volume of sequencing data for genotyping and apply the output toward the complex methods. In this way, the agronomic traits can be genetically mapped in a cost- and labor-effective manner.
